# *Funneliformis mosseae* Enhances Root Development and Pb Phytostabilization in *Robinia pseudoacacia* in Pb-Contaminated Soil

**DOI:** 10.3389/fmicb.2019.02591

**Published:** 2019-11-12

**Authors:** Li Huang, Deqiang Chen, Haoqiang Zhang, Yingying Song, Hui Chen, Ming Tang

**Affiliations:** ^1^State Key Laboratory of Conservation and Utilization of Subtropical Agro-Bioresources, Guangdong Key Laboratory for Innovative Development and Utilization of Forest Plant Germplasm, College of Forestry and Landscape Architecture, South China Agricultural University, Guangzhou, China; ^2^Food Science and Engineering, Beibu Gulf University, Qinzhou, China; ^3^College of Forestry, Northwest A&F University, Yangling, China

**Keywords:** arbuscular mycorrhizal fungi, *Funneliformis mosseae*, black locust, root characteristics, Pb-contaminated soil, phytostabilization

## Abstract

It is possible that arbuscular mycorrhizal fungi play a pivotal role in root development and Pb phytostabilization in plants grown in Pb-contaminated soil. In this study, a pot experiment was conducted over 4 months to evaluate the effects of *Funneliformis mosseae* strain BGCXJ01A on root characteristics of black locust (*Robinia pseudoacacia* L.) seedlings in Pb-contaminated soil. Four Pb treatments (0, 90, 900, and 3,000 mg kg^–1^) were applied to soil in the presence and absence of *F. mosseae*. Inoculation with *F. mosseae* prominently improved root length, surface area, volume, and tip number in the plants across all Pb treatments. The *F. mosseae* inoculation also increased root diameter and fork number, especially under high Pb treatments. The presence of *F. mosseae* significantly increased the root activity and root tolerance index. However, there was little difference in specific root length between inoculated and non-inoculated plants. The biomass of roots, stems, and leaves all increased following inoculation with *F. mosseae*. Inoculated plants had greater accumulation and translocation capacities for Pb in the roots and stems, but lower capacities were found in the leaves when compared with those in non-inoculated plants. These results highlight that *F. mosseae* can alleviate the toxic effects of Pb on root development and can immobilize Pb in the roots and stems of *R*. *pseudoacacia* grown in Pb-contaminated soil. This study provides a model system for phytoremediation of Pb-contaminated soil *via* reciprocal symbiosis between arbuscular mycorrhizal fungi and woody legumes.

## Introduction

Lead (Pb) is a non-essential toxic metal that exists in the majority of terrestrial ecosystems due to natural factors (such as the decay of thorium and uranium) and anthropic factors (such as industrial emissions and transport) (Rodríguez-Seijo et al., 2015). Metallurgy, energy production, and Pb-containing paints have been the primary sources of Pb in soil (Fahr et al., 2013). Due to its long retention time in the soil, Pb ultimately tends to accumulate in the food chain and generates a grievous threat to biota (Naik et al., 2012). Thus, the bioremediation of Pb-contaminated soils has become a global concern. Traditional physicochemical methods for soil remediation include electrochemical processes, burial, washing, thermal treatment, physical separation, and stabilization/solidification (Rajkumar et al., 2012). However, these methods are usually expensive and could result in damage to the soil structure accompanied by a reduction in soil bioactivity and nutrient depletion (Wang et al., 2010).

Phytoremediation is a cost-effective and environmentally friendly technology that uses plants and their associated microbes for the extraction, sequestration, or detoxication of heavy metals (HMs) from contaminated soils (Khan, 2005; Arshad et al., 2007; Jing et al., 2007; Saleem and Moe, 2014). Based on the different mechanisms used, phytoremediation can be divided into five major subgroups: (1) phytoextraction, using the roots of particular plants to take up toxic HMs from the soil and transport them to the harvestable parts; (2) phytodegradation, degrading HM-chelating compounds by plants and root-associated microbes such as arbuscular mycorrhizal (AM) fungi; (3) rhizofiltration, utilizing the plant roots to assimilate, enrich, and deposit HMs (such as Pb), which can remove a large amount of contaminants from sewage; (4) phytostabilization, immobilizing, reducing, or transforming HMs into less toxic forms using particular substances (such as root exudates and microbial metabolites) in the rhizosphere; and (5) phytovolatilization, transforming HMs (such as Se, As, and Hg) into a volatile state and then volatilizing them from the soil and plant surfaces (Khan, 2005; Arshad et al., 2007; Jing et al., 2007; Saleem and Moe, 2014).

Many HM-hyperaccumulator plants with resistance to single or multiple HMs have been reported, but most of them are characterized by low biomass and limited growth (Khan, 2005). To guarantee the feasibility of phytoremediation, which is a slow process, it is necessary to select fast-developing and HM-tolerant plants with widespread roots. However, plant uptake of HMs is constantly limited by their bioavailability in the soil. The rhizosphere microbiota can enhance the phytoremediation efficiency (Marques et al., 2009). In particular, AM fungi have been found in HM-contaminated soils where they constitute a crucial component of the rhizosphere microbiota and contribute to the revegetation of degraded habitats (Khan, 2005).

The AM fungi of the phylum Glomeromycota have existed for over 460 million years (Khan, 2005). The mutualistic symbiosis between AM fungi and plants is ubiquitous (He et al., 2016; Murugesan et al., 2016). In natural conditions, AM fungi are associated with at least 80% of terrestrial plants, including pteridophytes, gymnosperms, and angiosperms (Huang et al., 2017). Gildon and Tinker (1981) first reported an HM-tolerant strain of AM fungi (*Glomus mosseae*) that could increase HM uptake by clover (*Trifolium repens*) seedlings in soil cocontaminated with Zn and Cd. Meier et al. (2012) found that AM fungi could affect the distribution of HMs at the soil–fungi–plant interface, eventually alleviating their toxicity to plants. Following inoculation with AM fungi, various effects have been observed in phytoremediation. These effects include increasing the degradation efficiency of HMs and improving the establishment and development of plants (Khan, 2005). The AM fungi with abundant aseptate hyphae can be regarded as an extension of plant roots because they expand soil volume to strengthen plant water and nutrient uptake (Sheng et al., 2009). Additionally, the branched tree-like arbuscules of AM fungi facilitate the transportation of mineral nutrients (Gutjahr and Paszkowski, 2013). Mycorrhizal plants may increase the uptake of HMs and transfer them from roots to shoots (phytoextraction) or immobilize them in soil (phytostabilization) depending on factors such as the climate, soil properties, and the HM–plant–fungus combination (Mishra et al., 2017).

Successful phytoremediation mainly relies on the selection of plants that may potentially have a high biomass and great tolerance to contamination (Rajkumar et al., 2012). Black locust (*Robinia pseudoacacia* L.), a woody nitrogen-fixing legume, is widely distributed in semiarid regions across the world (He et al., 2016). Since the 1950s, *R*. *pseudoacacia* forests have been widespread on the Loess Plateau in China, where they enhance soil aggregate stability and maintain soil structure (Zhang et al., 2016). Importantly, *R*. *pseudoacacia* can survive and develop intimate symbioses with glomeromycotan fungi in HM-contaminated soils (Yang et al., 2015). The leaves of *R*. *pseudoacacia* are considered a biomonitor of HM contamination (Çelik et al., 2005). As a pioneer tree species, *R. pseudoacacia* is deemed to be a favorable candidate for the phytoremediation of HM-contaminated soils. *R. pseudoacacia* displays high stress tolerance, rapid plant growth, efficient nitrogen fixation, and deep root systems (Vlachodimos et al., 2013).

The root is a crucial organ for stabilization and nutrient uptake in plants. Moreover, the root plays a critical role in plant adaptation, health, and nutrition through phenotypic traits, such as root length, biomass, density, volume, and surface area (Saleem et al., 2018). Among other characteristics, the root plays a role in altering rhizosphere breadth, soil chemistry, and recruiting plant beneficial microbes (Saleem et al., 2018). Thus, it is critical for woody species to rapidly develop a functional root system (Berta et al., 1995). Alterations in the root architecture may be attributed to environmental factors including the presence of HMs (Keller et al., 2003; Fahr et al., 2013) and AM fungi (Sheng et al., 2009). AM fungi-induced root system remodeling is mediated by signal molecules from AM fungi, in addition to nutrient variation and distribution in the plant roots (Gutjahr and Paszkowski, 2013). To minimize metabolic costs during the establishment of fungi–plant symbioses, the root systems must be highly architecturally plastic and show appropriate responses and adaptation to the environment. This occurs mainly through lateral root proliferation (Gutjahr and Paszkowski, 2013). The development of branch roots could be facilitated by C reserves in the culture substrate (MacGregor et al., 2008). Since the plant root system is the primary pathway for the transfer of HMs into the food chain, it is important to investigate root development and HM distribution in hyperaccumulator plants. Currently, how AM fungi affect plant root systems during the phytostabilization process in Pb-contaminated soil is still poorly understood.

Herein, we assessed the possible effects of AM fungi on root characteristics and Pb accumulation in *R. pseudoacacia* seedlings grown in Pb-contaminated soil. A pot experiment was performed under different Pb treatments, with or without *Funneliformis mosseae* inoculation. The differences between inoculated and non-inoculated plants were then determined in terms of (1) root activity; (2) plant dry weight, root morphology, specific root length, and root tolerance index; and (3) plant Pb concentration, and Pb bioconcentration and translocation factors. This study will contribute to our understanding of root characteristics in woody legumes that are in mutualistic symbioses with AM fungi for the phytoremediation of Pb-contaminated soils.

## Materials and Methods

### Plant and Fungus

Seeds of *R. pseudoacacia* L. were collected from the campus of Northwest A&F University (Yangling, Shaanxi Province, China). Surface sterilization of seeds was performed with a 10% hydrogen peroxide solution for 10 min, followed by rinses with distilled water. The seeds were soaked in distilled water overnight and then pregerminated on BioClean filter paper (Wohua, Hangzhou, China) in Petri dishes (28°C for 2 days).

A commercial AM fungus, *F. mosseae* (BGCXJ01A), was provided by Beijing Academy of Agriculture and Forestry Sciences (Beijing, China). Spores of *F*. *mosseae* were germinated with *Zea mays* on clean fine sand in greenhouse conditions (35/20°C, day/night; relative humidity, 60%). Three months later, the mean mycorrhizal colonization was assessed (91.7%). The *F*. *mosseae* inoculant consisted of spores (∼26 spores g^–1^), external hyphae, and infected root segments in addition to the sand.

### Growth Substrate

Topsoil (0–30 cm) was collected from Northwest A&F University. The soil was air-dried and homogenized in a ceramic mill. The soil was then passed through a 2-mm sieve to remove stones and other debris. Subsequently, the soil was mixed with thoroughly washed fine sand at a sand-to-soil ratio of 1:2 (v/v). After autoclaving (at 121°C for 2 h), the basic properties of the mixture were analyzed according to the standard testing methods of Bao (2000). The substrate had a pH of 7.66 in a 1:2.5 (w/v) soil–water suspension ratio. The substrate contained (on a dry weight basis): 14.85 g kg^–1^ organic matter; 25.77 mg kg^–1^ nitrate-N, 7.37 mg kg^–1^ ammonium-N, 11.48 mg kg^–1^ available P, 128.96 mg kg^–1^ soluble K, and 6.58 mg kg^–1^ total Pb.

To prepare the Pb-contaminated growth substrate, the mixture of soil and sand was sprayed with lead nitrate (Pb[NO_3_]_2_) solutions to obtain gradient concentrations of Pb: 0 (control), 90, 900, and 3,000 mg Pb kg^–1^ soil. These concentrations were chosen based on the results of a preliminary experiment. The concentrations of Pb added corresponded to common levels of Pb contamination according to the soil environmental quality standard in China. The quantity of nitrate added as Pb(NO_3_)_2_ was offset by supplying a reduced amount of ammonium nitrate [NH_4_NO_3_]. After mixing thoroughly, the growth substrate was allowed to stabilize for 1 month before use.

### Experimental Procedure

A pot experiment was conducted in a conservatory over 4 months from March to July, 2014. Four different Pb treatments (0, 90, 900, and 3,000 mg kg^–1^ Pb) were applied in the presence or absence of *F. mosseae*. The experiment used a completely randomized factorial block design, with 30 replicates for each Pb treatment. Plastic pots (diameter = 10 cm, height = 8 cm) were filled with approximately 450 g of growth substrate each and divided into two groups. In the inoculated group, a small hole (diameter and depth = 3 cm) was dug at the surface of the substrate, followed by inoculation with 20 g of newly prepared *F*. *mosseae* inoculant. The non-inoculated group received 20 g of sterilized *F*. *mosseae* inoculant containing 10 mL of fungus-free leachate (pore size = 10 μm) from the *F*. *mosseae* spore suspension culture (sand:water = 1:10, w/v).

Following inoculation, four pregerminated seeds of uniform size were sown in each hole and covered with the substrate. Ten days after germination, one seedling was kept in each pot. All pots were maintained at room temperature under a natural illumination regimen throughout the experimental period: 35°C in the daytime and 20°C during the night. Soil humidity was measured with a TDR 100 tensiometer (Spectrum Technologies Inc., Chicago, IL, United States). To maintain the field capacity at a relatively stable level (∼60%), all pots were measured separately; water loss was recorded, and tap water was supplemented accordingly. Specifically, 35 mL of tap water was supplied to each pot every day, and 35 mL of 1/4 × fresh Hoagland’s nutrient solution was applied weekly right after preparation (Hoagland and Arnon, 1950) during the course of the whole growth period.

### Sampling and Analysis

Three seedlings aged 4 months old were randomly selected and harvested from each treatment group. The whole plants were gently washed with tap water, followed by rinses with deionized water and drying with paper towels. Then, the roots were separated from the aboveground parts and split into three groups: one to be used for the analysis of mycorrhizal colonization rate and root activity, one for the evaluation of root morphological characteristics, and one for the measurements of root biomass and Pb concentration. Additionally, the aboveground parts were divided into stems and leaves for Pb analysis.

The extraradical hyphae and spores of fresh roots were observed with an SZ2-ILST light- stereomicroscope at 25 × magnification (Olympus, Tokyo, Japan). Mycorrhizal colonization of fresh fine roots (0.5 g) was determined with a BX51 optical microscope at 200 × magnification (Olympus, Tokyo, Japan) as described by Phillips and Hayman (1970). The root activity of fresh fine roots (0.5 g) was measured using a 2,3,5-triphenyltetrazolium chloride assay (Sheng et al., 2009).

To characterize root morphology, the roots were soaked with tap water, evenly dispersed in root disks, and scanned with an Epson Expression 1680 Pro scanner (Epson, Nagano, Japan) at 300 dpi. Root parameters including total length (RL), volume (RV), surface area (RA), average diameter (RD), tip number (RT), and fork number (RF) were estimated using Win-RHIZO Pro version 2003b (Regent Instruments Inc., Canada). The specific root length (SRL) and the root tolerance index (RTI) were calculated using the following equations: SRL = root length/root dry weight; and RTI = root length in the treatment group/root length in the control group.

To desorb extracellular and apoplastic Pb, the roots were immersed in an ethylenediaminetetraacetic acid disodium solution (20 mM Na_2_EDTA) for 15 min. The roots were then dried at 70°C in an oven until a constant weight was reached. The dry weights of the roots, stems, and leaves were recorded. For the analysis of Pb in the plants, the subsamples of dried roots, stems, and leaves were ground and sieved through a 100-μm nylon mesh. The fine powder materials were digested in concentrated acids (HNO_3_:HClO_4_ = 4:1, v/v). The Pb concentration was measured using an AA-6300C flame atomic absorption spectrophotometer (Shimadzu, Kyoto, Japan) and expressed on a dry weight basis.

The Pb accumulation and tolerance in plants were evaluated using the bioconcentration factor (BCF) and translocation factor (TF), respectively, which were calculated as follows: BCF = plant Pb concentration/soil Pb concentration; and TF = shoot Pb concentration/root Pb concentration (Wei et al., 2012).

### Statistical Analysis

Values are presented as means of triplicate measurements ± standard deviation (SD). Data normality and homogeneity of variance were examined using the Kolmogorov-Smirnov test and Levene test, respectively. All of the raw data sets conformed to a Gaussian distribution. Possible differences in group means among the Pb treatments were assessed using a one-way analysis of variance (ANOVA), and significant differences were identified at *P* < 0.05 using Duncan’s multiple range test. An independent-sample *t*-test was used to analyze the significant variations in root characteristics between the plants with and without *F*. *mosseae* inoculation for each Pb treatment. Statistical analyses were completed using SPSS 22.0 (IBM SPSS, Somers, United States), and diagrams were drawn using SigmaPlot 10.0 (Systat Software, San Jose, CA, United States).

## Results

### Mycorrhizal Colonization

**FIGURE 1:**
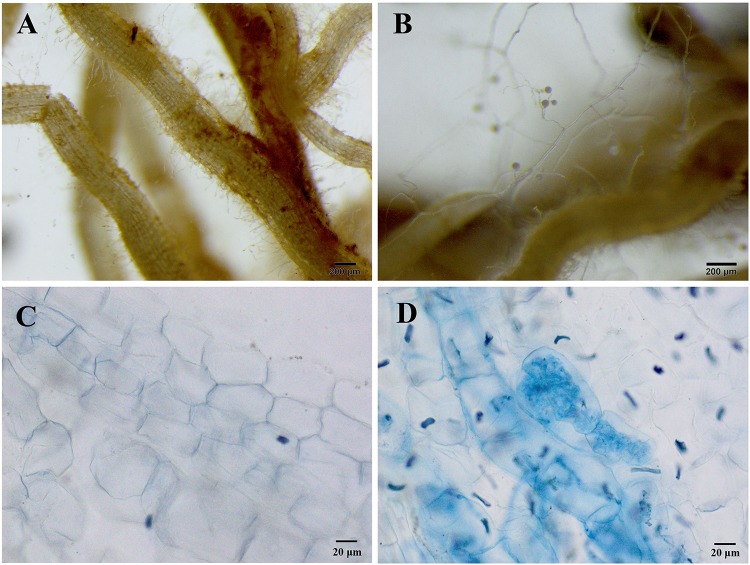
Microphotographs showing the symbiotic relationships between *Funneliformis mosseae* and *Robinia pseudoacacia* seedlings grown under the control Pb treatment (0 mg Pb kg^– 1^ soil) for 4 months. **(A)** Roots of non-inoculated plants without any extraradical hyphae and spores. **(B)** Roots of inoculated plants with extraradical hyphae and spores. **(C)** Root cells of non-inoculated plants. **(D)** Intraradical hyphae and arbuscular in the root cells of inoculated plants. **(A,B)** 25 × magnification; **(C,D)** 200 × magnification.

**FIGURE 2:**
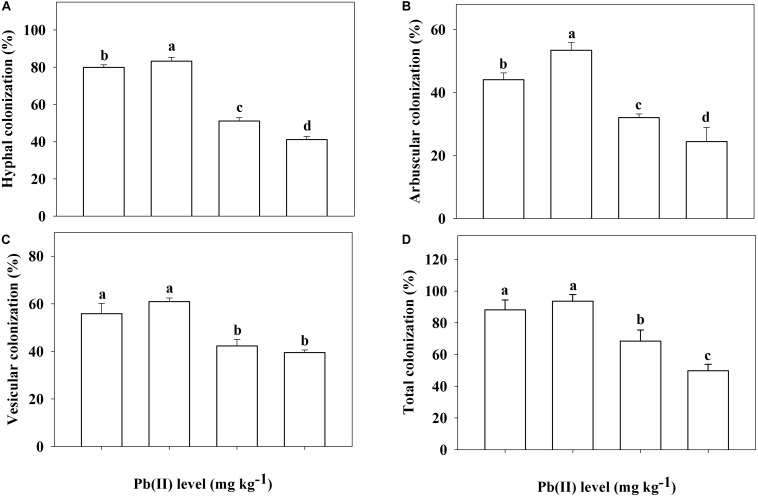
Root colonization of *Robinia pseudoacacia* seedlings by *Funneliformis mosseae* being subjected to different Pb levels for 4 months. **(A)** Hyphal colonization; **(B)** Arbuscular colonization. **(C)** Vesicular colonization. **(D)** Total colonization. Shown are means ± SD (*n* = 3). Different lowercase letters indicate significant differences among different Pb levels within the inoculated treatment (*P* < 0.05; ANOVA with *post hoc* Duncan).

In the non-inoculated plants, root colonization of *R. pseudoacacia* seedlings by the inoculum *F. mosseae* was not observed (Figures 1A,C). In the inoculated plants without Pb addition, the mutualistic symbiosis between *F*. *mosseae* and *R*. *pseudoacacia* was evident. There were fungal hyphae and spores outside the roots and arbuscules inside the roots (Figures 1B,D). Four months after inoculation, the hyphal, arbuscular, vesicular, and total colonization rates in plants treated with 0 mg kg^–1^ Pb were 79.9, 44.1, 55.9, and 88.2%, respectively. With increasing Pb level, the hyphal, arbuscular, vesicular, and total colonization rates all decreased substantially. The lowest colonization rates (41.2, 24.4, 39.5, and 49.8%, respectively) were observed in plants treated with 3,000 mg kg^–1^ Pb (Figure 2).

### Root Activity and Root Morphology

**FIGURE 3:**
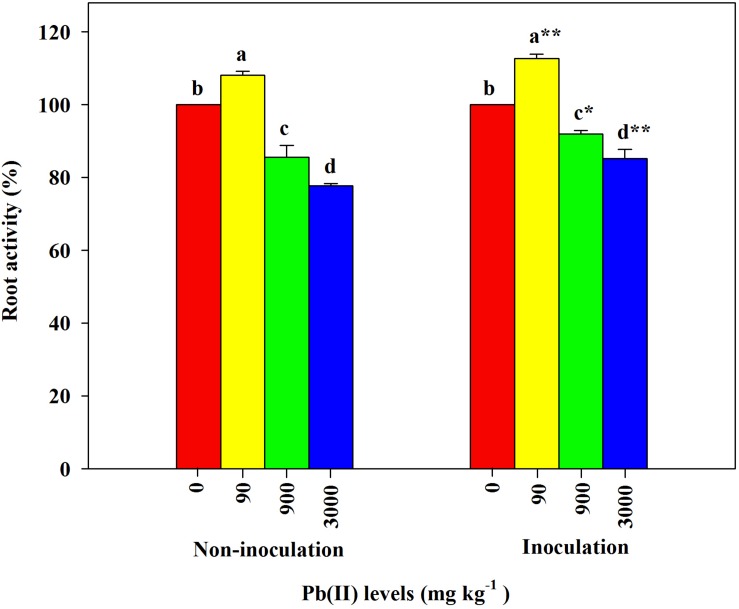
Relative changes in root activity of *Robinia pseudoacacia* seedlings grown with or without *Funneliformis mosseae* and subjected to different Pb levels for 4 months. Shown are means ± SD (*n* = 3). Asterisks indicate significant differences between inoculated and non-inoculated seedlings within each Pb treatment (^∗^*P* < 0.05, ^∗∗^*P* < 0.01; *t*-test). Different lowercase letters indicate significant differences among different Pb levels within inoculated or non-inoculated treatment (*P* < 0.05; ANOVA with *post hoc* Duncan).

Under the control conditions (0 mg kg^–1^ Pb), there were significant differences in the root activity of *R. pseudoacacia* seedlings between inoculated and non-inoculated plants. There was a 9.8% increase in the former compared with the latter group (Table 1). Compared with the controls, the root activity of both inoculated and non-inoculated plants significantly increased under 90 mg kg^–1^ Pb (Figure 3). A larger increase in root activity was found in inoculated plants than that in non-inoculated plants (13 versus 8%). In contrast, pronounced decreases in root activity occurred under Pb levels of 900 and 3,000 mg kg^–1^. The decrease was smaller for inoculated plants relative to non-inoculated plants (15 versus 22% under 3,000 mg kg^–1^ Pb).

**TABLE 1:** Root activity and morphological parameters of *Robinia pseudoacacia* seedlings inoculated with or without *Funneliformis mosseae* and grown under the control Pb treatment (0 mg Pb kg^–1^ soil) for 4 months.

**Parameter**	**Non-inoculation**	**Inoculation**
Root activity (μg g^–1^ h^–1^)	156.02	171.32^∗∗^
Root length (cm)	3,134.62 ± 242.16	4,798.53 ± 662.27^∗^
Root surface (cm^2^)	398.95 ± 23.84	718.18 ± 67.13^***^
Root volume (cm^3^)	4.27 ± 0.37	5.72 ± 0.99^NS^
Number of root tips	16,533.33 ± 175.25	33,782.67 ± 3,271.34^***^
Root diameter (mm)	0.33 ± 0.02	0.45 ± 0.02^***^
Number of root forks	35,009.67 ± 2627.45	40,368.33 ± 6,970.8^NS^
Specific root length (cm g^–1^)	1,575.23 ± 121.47	2,111.67 ± 261.45^∗^

*Shown are means ± SD (*n* = 3). Asterisks indicate significant differences between inoculated and non-inoculated groups under 0 mg kg^–1^ Pb (^∗^*P* < 0.05, ^∗∗^*P* < 0.01, ^∗∗∗^*P* < 0.000; *t*-test). NS: no significance.*

**FIGURE 4:**
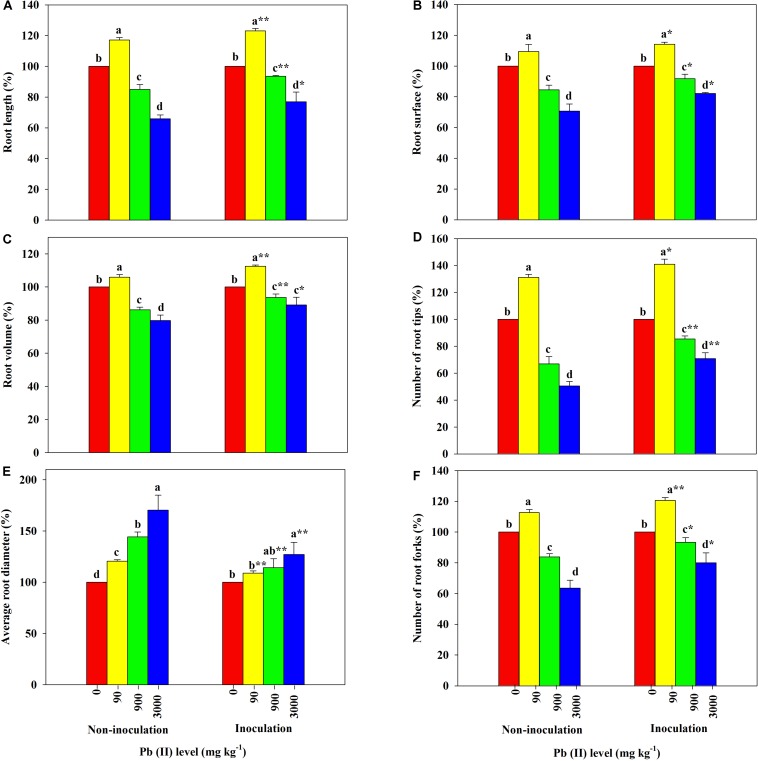
Relative changes in root morphology of *Robinia pseudoacacia* grown with or without *Funneliformis mosseae* and subjected to different Pb levels for 4 months. **(A)** Root length. **(B)** Root surface area. **(C)** Root volume. **(D)** Number of root tips. **(E)** Average root diameter. **(F)** Number of root forks. Shown are means ± SD (*n* = 3). Asterisks indicate significant differences between inoculated and non-inoculated seedlings within each Pb treatment (^∗^*P* < 0.05, ^∗∗^*P* < 0.01; *t*-test). Different lowercase letters indicate significant differences among different Pb levels within inoculated or non-inoculated treatment (*P* < 0.05; ANOVA with *post hoc* Duncan).

The root morphological parameters of *R. pseudoacacia* seedlings across different treatments are shown in Figure 4. The RL, SA, RV, RT, and TF were all greater in plants treated with 90 mg kg^–1^ Pb relative to the controls; however, further increases in the Pb level led to obvious decreases in the parameter values. Additionally, the RD in all treated plants increased with increasing Pb level. Among these results, the RL, SA, RV, RT, and TF of inoculated plants decreased less than those of non-inoculated plants across all Pb treatments. For example, the RL, SA, RV, RT, and TF decreased by 23 versus 34%, 18 versus 29%, 11 versus 20%, 29 versus 49%, and 20 versus 36%, respectively, under 3,000 mg kg^–1^ Pb. The variation in the RD of inoculated plants was much smaller than that of non-inoculated plants across different Pb levels (27 versus 70% under 3,000 mg kg^–1^ Pb).

### Plant Biomass

The dry weights of roots, stems, and leaves for all *R. pseudoacacia* seedlings are summarized in Table 2. When treated with low Pb of 90 mg kg^–1^, a distinctly increasing trend was observed in the biomass of all plant tissues, with the exception of leaves in non-inoculated plants. Under medium to high Pb levels of 900 and 3,000 mg kg^–1^, plant biomass clearly decreased in all plant tissues compared with those in the respective controls. For example, the plant biomass decreased by 24.1 and 40.2% in the roots of non-inoculated plants under 900 and 3,000 mg kg^–1^ Pb, respectively. For the same Pb treatment, the biomass of inoculated plants was always higher than that of non-inoculated plants (18.4% higher in leaves under 3,000 mg kg^–1^ Pb).

**TABLE 2:** Plant dry weight, Pb concentrations, and bioconcentration factor of Pb in various tissues of *Robinia pseudoacacia* seedlings in response to different Pb levels (0, 90, 900, and 3,000 mg Pb kg^–1^ soil) for 4 months.

**Plant**	**Pb(II) level**		**Dry weight**	**Plant Pb**	**Bioconcentration**
**tissue**	**(mg kg^–1^)**	**AMF**	**(g plant^–1^)**	**(mg kg^–1^, DW)**	**factor**
Roots	0	−M	1.99 ± 0.03c	0.61 ± 0.03g	0.09 ± 0.01f
		+M	2.27 ± 0.06b	0.56 ± 0.03g	0.08 ± 0.01f
	90	−M	2.44 ± 0.11b	78.98 ± 0.56f	0.88 ± 0.02b
		+M	2.87 ± 0.28a	101.44 ± 2.16e	1.13 ± 0.02a
	900	−M	1.51 ± 0.15de	253.81 ± 9.91d	0.28 ± 0.02d
		+M	1.72 ± 0.02de	496.69 ± 5.96c	0.56 ± 0.02c
	3,000	−M	1.19 ± 0.02f	666.93 ± 24.51b	0.22 ± 0.02e
		+M	1.37 ± 0.06ef	810.77 ± 9.98a	0.27 ± 0.02d
Stems	0	−M	0.58 ± 0.02d	0.25 ± 0.02f	0.04 ± 0.01g
		+M	0.66 ± 0.02c	0.19 ± 0.02f	0.03 ± 0.01g
	90	−M	0.78 ± 0.03b	27.60 ± 1.11e	0.31 ± 0.01b
		+M	0.85 ± 0.03a	29.15 ± 1.45e	0.32 ± 0.01a
	900	−M	0.51 ± 0.01e	79.09 ± 1.43d	0.09 ± 0.01d
		+M	0.56 ± 0.01d	118.66 ± 5.54c	0.13 ± 0.01c
	3,000	−M	0.32 ± 0.01f	158.28 ± 6.21b	0.05 ± 0.01f
		+M	0.33 ± 0.01f	182.02 ± 3.23a	0.07 ± 0.01e
Leaves	0	−M	2.16 ± 0.03c	0.14 ± 0.01f	0.02 ± 0.01e
		+M	2.33 ± 0.02b	0.13 ± 0.02f	0.02 ± 0e
	90	−M	2.29 ± 0.01bc	18.61 ± 1.15e	0.21 ± 0.01a
		+M	2.48 ± 0.16a	16.33 ± 1.40e	0.18 ± 0.01b
	900	−M	1.35 ± 0.11e	50.84 ± 2.87c	0.06 ± 0.01c
		+M	1.55 ± 0.06d	39.99 ± 1.67d	0.04 ± 0d
	3,000	−M	1.14 ± 0.06f	101.72 ± 2.01a	0.03 ± 0.01d
		+M	1.35 ± 0.04e	66.08 ± 4.19b	0.02 ± 0.01e

*AMF, arbuscular mycorrhizal fungus *Funneliformis mosseae*; +M, inoculated group; −M, non-inoculated group. Shown are means ± SD (*n* = 3). Different letters within each group indicate significant differences among different Pb levels between inoculated and non-inoculated treatment (*P* < 0.05; ANOVA with *post hoc* Duncan).*

### Root Tolerance and Specific Length

**FIGURE 5:**
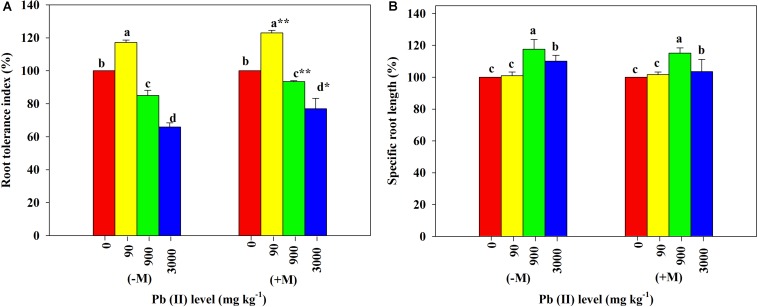
Relative changes in root tolerance index and specific root length of *Robinia pseudoacacia* seedlings grown with or without *Funneliformis mosseae* and subjected to different Pb levels for 4 months. **(A)** Root tolerance index. **(B)** Specific root length. Shown are means ± SD (*n* = 3). Asterisks indicate significant differences between inoculated and non-inoculated seedlings within each Pb treatment (^∗^*P* < 0.05, ^∗∗^*P* < 0.01; *t*-test). Different lowercase letters indicate significant differences among different Pb levels within inoculated or non-inoculated treatment (*P* < 0.05; ANOVA with *post hoc* Duncan).

Low Pb significantly increased the RTI in both inoculated and non-inoculated *R. pseudoacacia* seedlings compared with those in their respective controls. The increase of RTI in the inoculated plants was significantly greater than that in the non-inoculated plants. In contrast, high Pb significantly reduced the RTI in all plants with or without inoculation. The inoculated plants had significantly smaller relative reduction in their RTI relative to the non-inoculated plants (85 versus 94% under 900 mg kg^–1^ Pb and 66 versus 77% under 3,000 mg kg^–1^ Pb; Figure 5A).

Under the control conditions, the inoculated plants yielded the SRL of 2,111.67 cm g^–1^ on average, which was substantially higher than the average of non-inoculated plants (Table 1). With increasing Pb level, the SRL values of both inoculated and non-inoculated plants significantly increased (2,143.59 cm g^–1^ and 1,588.36 cm g^–1^ under 90 mg kg^–1^ Pb versus 2,436.06 cm g^–1^ and 1,856.12 cm g^–1^ under 900 mg kg^–1^ Pb). However, the relative increases in SRL did not differ significantly between plants with and without inoculation (Figure 5B).

### Plant Pb Concentration

The Pb concentrations in different tissues of all *R. pseudoacacia* seedlings significantly increased with increasing Pb level (Table 2). Under 900 and 3,000 mg kg^–1^ Pb, the inoculated plants had significantly higher root and stem Pb concentrations compared with those in the non-inoculated plants (by 95.7 and 21.6% in roots, respectively). In contrast, the corresponding leaf Pb concentrations in inoculated plants were significantly lower (*P* < 0.05) than those in non-inoculated plants under high Pb level (35.0% lower under 3,000 mg kg^–1^ Pb).

### Bioconcentration and Translocation of Pb

Compared with the respective controls, the BCF values in different plant tissues first increased under low Pb level and then decreased toward higher Pb levels (Table 2). The BCF values in the roots were higher than those in the stems and leaves for both inoculated and non-inoculated plants. Under the same Pb level, the inoculated plants had significantly higher BCF values in the roots and stems compared with those in non-inoculated plants. For example, under 900 mg kg^–1^ Pb, the BCF value in the roots was 100.0% higher in the inoculated plants than that in non-inoculated plants. However, under the same Pb level, the inoculated plants had lower BCF values in the leaves compared with those in non-inoculated plants. For example, under 900 mg kg^–1^ Pb, the leaf BCF value was 33.3% lower in inoculated plants.

**FIGURE 6:**
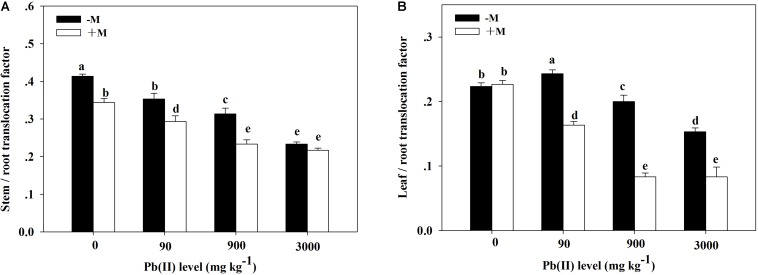
Translocation factor of Pb in *Robinia pseudoacacia* seedlings grown with or without *Funneliformis mosseae* and subjected to different Pb levels for 4 months. **(A)** Stem/root translocation factor. **(B)** Leaf/root translocation factor. Shown are means ± SD (*n* = 3). Different lowercase letters indicate significant differences among different Pb levels in inoculated or non-inoculated treatment (*P* < 0.05; ANOVA with *post hoc* Duncan).

The TF values showed obvious variation across the different treatments (Figure 6). The stem/root TF values showed a decreasing trend with increasing Pb level; the lowest values appeared under 3,000 mg kg^–1^ Pb. The leaf/root TF values in the non-inoculated plants first peaked under 90 mg kg^–1^ Pb (TF = 0.24) and then substantially decreased with increasing Pb level. However, in the inoculated plants, the leaf/root TF values markedly and continuously decreased with increasing Pb level. Generally, inoculated plants had lower stem/root and leaf/root TF values compared with those in non-inoculated plants across the different Pb treatments (for example, 0.23 versus 0.31 for the stem/root TF under 900 mg kg^–1^ Pb).

## Discussion

In this study, Pb-tolerant *R*. *pseudoacacia* seedlings were grown in pots with or without the AM fungus *F*. *mosseae* and subjected to different Pb treatments. Based on the analysis of root characteristics and the Pb distribution in plant tissues, we investigated the effects of *F*. *mosseae* inoculation on root development and Pb phytostabilization in *R*. *pseudoacacia* seedlings under Pb stress. The results highlight the possibility of using *F*. *mosseae* to alleviate soil Pb toxicity and enhance Pb immobilization in *R*. *pseudoacacia*. Since plant roots are a crucial organ for the uptake of HMs and nutrients from soil, this study provides a model system for the phytoremediation of Pb-contaminated soil *via* reciprocal symbiosis between AM fungi and woody legumes.

Plant health can be affected by soil HMs *via* direct or indirect mechanisms (Mishra et al., 2017). In the present study, high Pb levels (≥900 mg kg^–1^) substantially reduced root activity in *R*. *pseudoacacia* seedlings. Similar results have been reported in maize seedlings by Liu et al. (2014). The root system interacts with HMs in the soil and modulates its morphological characteristics and physiological functions to maintain assimilation and subsistence (Fahr et al., 2013). As the root is the foremost organ of the plant to receive HM ions in the soil, the sensitivity of the roots to HMs can be much higher than that of aboveground plant parts (Lu et al., 2013). Root morphological characteristics such as RL, RS, RD, and RV may differentially affect plant adaptation, health, and productivity (Saleem et al., 2018). This is because these characteristics are closely associated with plant uptake of water and nutrients (Ding et al., 2014). However, in HM-contaminated soils, root development can be affected by the toxicity of the HMs to the plant. The inhibition of root elongation is taken as the first evidence of any adverse effects (Munzuroglu and Geckil, 2002). Direct contact between plant roots and HMs can lead to changes in the morphological traits and physiological functions of the roots (Mishra et al., 2017). Here, the decreases in the values of the root morphological parameters including RL, RS, RD, and RV in *R*. *pseudoacacia* seedlings were in accordance with the reduced root activity observed under high Pb treatments. Such root modifications were most likely associated with alterations in plant metabolism.

Microbial interactions in the soil can facilitate plant growth and function for ecosystem health and productivity (Saleem and Moe, 2014). In particular, fungi play a role in improving plant growth and stress tolerance in harsh environments (Chen et al., 2017; Qin et al., 2017). The AM fungi constitute one of the most prominent groups of soil fungi, and they can colonize the root cortex in most plant species (He et al., 2016). In the current study, the results showed that *F*. *mosseae* could establish symbioses with *R*. *pseudoacacia* in Pb-contaminated soil. The AM fungus successfully colonized the roots of *R*. *pseudoacacia* seedlings under different Pb treatments. Root colonization by AM fungi may be either tightly related to Andrade et al. (2004), Chen et al. (2005) or relatively unaffected by Liu et al. (2005) HM concentrations in the soil. Here, it was found that *F*. *mosseae* colonization was stimulated at a low Pb level (90 mg kg^–1^) but was substantially inhibited under higher Pb levels (≥900 mg kg^–1^). This decreased colonization may be attributed to the sensitivity of AM fungi to higher HM concentrations in the soil (Andrade et al., 2004). Similar results have been reported in several plant species including *Kummerowia striata* (Thunb.) Schindl and *Lolium perenne* L. (Chen et al., 2005).

Mycorrhizal symbioses can contribute to growth stimulation and higher HM uptake in plants under HM stress (Galli et al., 2010). Application of HM-tolerant AM fungi could assist host plants with growth regulation, root development (Berta et al., 1995), and HM accumulation (Chen et al., 2005). In the present study, the *R*. *pseudoacacia* seedlings inoculated with *F. mosseae* yielded higher biomass in various tissues (leaves, stems, and roots) compared with those in the non-inoculated plants across different Pb treatments. The positive effects of AM fungi on host plant growth may be attributed to the following factors: improved uptake of soil nutrients (such as P) (Wu et al., 2016), adjusted balance of endogenous plant hormones (such as cytokinins and gibberellins) (Liu et al., 2016), and the enhanced release of root exudates (such as polysaccharides) (Zangaro et al., 2005). The growth stimulation may have also been related to a high metabolic activity in the affected roots (Langer et al., 2010), which is in accordance with the higher root activity observed in the inoculated seedlings.

Early colonization by AM fungi is beneficial to the root morphology and performance of mycorrhizal seedlings (Berta et al., 1995). The AM associations could induce modifications in the root system in a textural, dimensional, quantificational, and impermanent manner (Sheng et al., 2009). For instance, Wu et al. (2011) have indicated that the mycorrhizal seedlings of *Poncirus trifoliata* have a greater RL, RA, and RV but a smaller RD than those in non-mycorrhizal plants. The RL is relevant to the plant’s capacity to uptake water and nutrients from the soil (Di Salvatore et al., 2008). In the current study, the RL of *R*. *pseudoacacia* seedlings was longer in plants inoculated with *F. mosseae* than that in non-inoculated plants. This result suggests a remission effect of AM fungi on the depolymerization of cytoskeletal structures and chromosome aberrations (Liu et al., 1994) and indicates that mycorrhizal plants have a higher capacity for resource acquisition (Wu et al., 2016). In adverse environments (such as those with salt stress, high temperature, or drought stress), AM fungi influence root morphology and play a positive role in plant growth. Similarly, endomycorrhizal symbiosis can primarily function to protect the root system of plants from HMs (Galli et al., 2010). Generally, the presence of HMs can facilitate the formation of shorter yet thicker main and lateral roots in plants grown in contaminated soils (Arduini et al., 1995). However, in the present study, a low Pb concentration (90 mg kg^–1^ Pb) led to increases in the RL, SA, RV, RT, and TF in *R*. *pseudoacacia* seedlings compared with those in the control plants grown without Pb. It is possible that this growth promotion effect is related to a stimulated metabolism (such as photosynthesis) or enzyme activities (such as superoxide dismutase) under low Pb treatment (Schützendübel and Polle, 2002).

The RD can respond to changes in soil physical conditions (Atkinson, 2000), and roots with a larger relative RD tend to have higher penetration capacity (Materechera et al., 1992). Wu et al. (2011) demonstrated that *F*. *mosseae* and *Glomus versiforme* inoculations appeared to reduce the RD in *Poncirus trifoliata* seedlings. However, in the present study, *F. mosseae* inoculation increased the RD in *R*. *pseudoacacia* seedlings relative to the non-inoculated plants under different Pb treatments. Similarly, Zangaro et al. (2007) observed a positive correlation between AM colonization and rootlet diameter in 12 local arboreal plants in both fertile and infertile soils of south Brazil. The larger RD observed in *F. mosseae*-inoculated plants under Pb stress was probably due to an increased parenchyma cell size, and accrescent cortical tissues resulted from the infection by AM fungi with increasing resistance to adverse habitat (Sheng et al., 2009; Lu et al., 2013). An increased density of root tips indicates more intensive exploitation of the substrate. Here, it was found that the RT in *R*. *pseudoacacia* seedlings decreased under Pb stress, irrespective of *F. mosseae* inoculation. The decreased RT under the Pb treatments suggests a diminishing capacity of *R*. *pseudoacacia* seedlings to acquire resources because the root tips are the closest part of the plant to free Pb^2+^ ions in the soil (Lu et al., 2013). However, the inoculated seedlings had a greater RT than that in non-inoculated seedlings, suggesting that *F*. *mosseae* could alleviate the reduction in lateral root proliferation and resource acquisition capacity.

Moreover, *F*. *mosseae* inoculation increased the root biomass and root Pb concentration in *R*. *pseudoacacia* seedlings when compared with those in the non-inoculated seedlings. With increasing Pb concentration, *F*. *mosseae* inoculation promoted plant growth and Pb uptake possibly by facilitating P uptake and mitigating Pb toxicity, with more Pb segregation in the roots (Chen et al., 2005; Andrade et al., 2010). The RA can affect the uptake efficiency in plants (Keller et al., 2003). Therefore, a plausible reason for the increased biomass and Pb uptake is that the presence of the external hyphae of *F*. *mosseae* expanded the RA of *R*. *pseudoacacia* seedlings, through which soluble mineral nutrients (especially P) could be assimilated (Andrade et al., 2010). However, non-inoculated seedlings display hindered and slow root development even with proper mineral (such as P) nutrition in the soil (Andrade et al., 2010).

Plant Pb analysis revealed that *F*. *mosseae* inoculation reduced Pb concentrations in the leaves while increasing Pb concentrations in the stems and roots of *R*. *pseudoacacia* seedlings compared with those in non-inoculated plants. This result indicates a differential Pb distribution in various tissues of plants that are grown with or without *F*. *mosseae*. Additionally, there were significant differences in Pb accumulation between inoculated and non-inoculated seedlings. The BCF values indicate the ability of plants to accumulate HMs (Wei et al., 2012). Under the same Pb treatment, *F. mosseae* inoculation obviously increased BCF values in the roots and stems but reduced BCF values in the leaves of *R*. *pseudoacacia* seedlings when compared with those in non-inoculated plants. Moreover, *F. mosseae* inoculation resulted in lower stem/root and leaf/root TF values in *R*. *pseudoacacia* seedlings. These results imply that the association of *F. mosseae* with *R*. *pseudoacacia* could enhance Pb phytostabilization in Pb-contaminated soil.

## Conclusion

The present study demonstrated the beneficial effects of *F*. *mosseae* inoculation on plant growth (especially root development) and Pb phytostabilization in *R*. *pseudoacacia* seedlings subjected to Pb stress. Compared with non-inoculated plants, inoculated plants yielded higher biomass, root activity, root morphological parameters (including RL, RA, RV, RD, RT, and RF), tolerance indices, and specific root lengths across the different Pb treatments. Moreover, *F*. *mosseae* inoculation increased Pb immobilization in the roots and stems but decreased Pb concentration in the leaves of *R*. *pseudoacacia* seedlings. These results indicate that *F*. *mosseae* may convey *R*. *pseudoacacia* with a higher tolerance and Pb uptake capacity in Pb-contaminated soil. The inoculated plants had better root development, higher biomass yield, and more Pb accumulation in particular tissues when subjected to Pb stress. It is necessary to explore whether root exudates or plant hormones from *R*. *pseudoacacia* seedlings participate in their responses to Pb stress.

## Data Availability Statement

All datasets generated for this study are included in the article/supplementary material.

## Author Contributions

LH and YS conducted the experiments and collected the data. LH, DC, and HZ drafted and revised the manuscript. HC, HZ, DC, and YS participated in data set assessment, script preparation, and manuscript revision. MT was the principal investigator who designed the study and finalized the script.

## Conflict of Interest

The authors declare that the research was conducted in the absence of any commercial or financial relationships that could be construed as a potential conflict of interest.
